# Severe vitamin B12 deficiency in an exclusively breastfed 5-month-old Italian infant born to a mother receiving multivitamin supplementation during pregnancy

**DOI:** 10.1186/1471-2431-12-85

**Published:** 2012-06-24

**Authors:** Sophie Guez, Gabriella Chiarelli, Francesca Menni, Simona Salera, Nicola Principi, Susanna Esposito

**Affiliations:** 1 Pediatric Clinic 1, Università degli Studi di Milano, Fondazione IRCCS Ca’ Granda Ospedale Maggiore Policlinico, Via Commenda 9, Milano, 20122, Italy

**Keywords:** Anemia, Iron deficiency, Neurological problems, Vegan diet, Vegan mothers, Vitamin B12 deficiency

## Abstract

**Background:**

In infants, vitamin B12 deficiency may be due to an inborn error of absorption and metabolism, or nutritional problems.

**Case presentation:**

An exclusively breastfed 5-month-old Italian male infant, who was born after a normal full-term pregnancy to a vegan mother who was apparently daily treated with a multivitamin oral preparation during the second and third trimester, was hospitalised because of poor weight gain, feeding difficulties, severe pallor, muscle hypotonia and somnolence. Upon admission, his weight, length and head circumference were below the third percentile, he had an enlarged liver and spleen, and showed a significant delay in developmental milestones and communicative reactions. He had a hemoglobin level of 4.7 g/dL with an MCV of 84.2 fL, a white blood cell count of 4,680/mm^3^, and a platelet count of 45,000/mm^3^. His serum vitamin B12 level was 57 pg/mL (normal value 180–500 pg/mL) and serum folate level 12.8 ng/mL (normal value >3 ng/mL). The results of metabolic examinations excluded a cobalamin C disorder, whereas nutritional screening showed a serum iron concentration of 9 μg/dL and serum ferritin of 4 ng/mL. Magnetic resonance imaging of the brain showed mild dilatation of the lateral ventricles with diffuse delayed myelination. The child was diagnosed as having vitamin B12 and iron deficiency due to nutritional inadequacy and was immediately treated with packed red blood cells, intramuscular vitamin B12 injections, and iron supplementation. A few days after the start of therapy, his hemoglobin levels and other hematological parameters rapidly improved, and a clinical improvement was observed within few weeks. There was an increase in his achievement of developmental milestones, but his development was still retarded seven months after the start of therapy.

**Conclusion:**

This case underlines the importance of adequately controlling maternal vitamin B12 intake during pregnancy by means of supplementation which, in the case of vegan mothers, should be significantly greater than that usually given. Moreover, the supplementation should be continued during lactation in order to avoid the development of signs of deficiency that may be associated with persistent neurological problems in infants. The case also highlights the need to consider vitamin B12 deficiency in infants with severe anemia even if their hematological parameters do not indicate megaloblastic anemia because the concomitant presence of substantial iron deficiency may modify the characteristics of the anemia.

## Background

Vitamin B12 plays a major role in human intermediary metabolism. It is required to convert methylmalonyl-CoA to succinyl-CoA (a compound metabolised by the Krebs cycle to produce energy) and to ensure the activity of methionine synthase, an enzyme that catalyses the methylation of homocysteine to form the essential amino acid methionine [1].

Vitamin B12 deficiency leads to the accumulation of methylmalonic acid and homocysteine in blood and urine, and the onset of clinical hematological, neurological and psychiatric manifestations [2]. Vitamin B12 deficiency in infancy may be due to an inborn error of absorption and metabolism, or (more frequently) nutritional problems. The most frequent inborn error is cobalamin C disorder, which is caused by a mutation of the *MMACHC* gene encoding a protein that plays a critical role in the metabolic pathway leading to the formation of succinyl-CoA and methionine [3]. However, most infants with B12 deficiency are born to women with low vitamin B12 levels and have been exclusively breastfed. Vitamin B12 is only found in animal products such as meat, egg, fish and milk [4]. Consequently, the breast milk of mothers who do not consume such products is frequently poor in vitamin B12, and their newborn infants have low vitamin stores.

We describe the case of an exclusively breastfed 5-month-old Italian infant with severe pancytopenia and neurological impairment who was born to a vegan mother who had received B12 supplementation during pregnancy but not during lactation.

## Case presentation

This five-month-old male Italian infant was born after a normal full-term (41 weeks) pregnancy with a weight of 2,550 g (3rd percentile), length of 48 cm (10^th^ percentile) and head circumference of 35 cm (25^th^ percentile). His mother, who had been vegan for several years, was apparently daily treated with a multivitamin oral preparation with a multivitamin preparation (Multicentrum, Pfizer Consumer Healthcare, Rome, Italy) during the second and third trimester in order to ensure an intake of 2.5 μg of vitamin B12 per day. Unfortunately, this supplementation was stopped after delivery. The child was exclusively breastfed until the fifth month of life, when he was hospitalised because of poor weight gain, feeding difficulties, severe pallor, muscle hypotonia and somnolence. The parents reported that some of these signs had been present from the third month of life. The child was seen by a paediatrician at the age of one month and three months, and physical evaluation resulted normal. Physical examination upon admission confirmed the pallor and revealed that the child was below the 3rd percentile for weight (4,800 g), length (60 cm) and head circumference (39 cm), had an enlarged liver and spleen, showed a significant delay in the developmental milestones and communicative reactions for his age. He had a hemoglobin level of 4.7 g/dL with an MCV of 84.2 fL, a white blood cell count of 4,680/mm^3^ and neutrophil count of 1,900 mm^3^. His platelet count was 45,000/mm^3^. The peripheral blood film showed oval macrocytes, anisopoikilocytosis, hypochromia, anisochromia and hyper-segmented polymorphonuclear leukocytes. His serum vitamin B12 level was 57 pg/mL (normal value 180–500 pg/mL) and serum folate level 12.8 ng/mL (normal value >3 ng/mL). Blood homocysteine was 11 μmol/L (normal value 4–15 μmol/L) and urinary methylmalonic acid 281 mmol/moL creatinine (normal value <5 mmol/moL creatinine). No intrinsic factor antibodies were found. Hormonal and nutritional screening revealed no other deficiency except for a serum iron concentration of 9 μcg/dL (normal value >29 μg/dL) and serum ferritin of 4 ng/mL (normal value 20–270 ng/mL). Thalassemia screening resulted negative. A bone marrow examination showed a reduced number of red line cells with some megaloblasts, a small number of dysplastic megakaryocytes, and dysplastic granulopoiesis with asynchronous maturation processes. Genetic analyses did not find any *MMACHC* gene mutations.

Magnetic resonance imaging (MRI) of the brain showed mild dilatation of the lateral ventricles with diffuse delayed myelination that was most marked in the brainstem.

Maternal investigations showed a hemoglobin level of 9.1 g/dL with an MCV of 96.4 fL, and serum vitamin B12 level of 155 pg/mL (no data on maternal serum vitamin B12 during pregnancy was available).

On the basis of these data, the child was diagnosed as having vitamin B12 and iron deficiency due to nutritional inadequacy. He was immediately treated with packed red blood cells, intramuscular vitamin B12 injections (at a dose of 1,000 μg/day for two weeks followed by weekly injections at the same dose for six months) and oral iron supplementation (iron sulphate 3 mg/kg/day for 3 months).

Table 1 shows the patient’s clinical and laboratory data upon admission and during follow-up. A few days after the start of therapy, his hemoglobin levels and other hematological parameters rapidly improved, and a clinical improvement was observed after a few weeks. The patient was discharged after 19 days.

**Table 1:** Admission and follow-up clinical and laboratory data of an infant with vitamin B12 deficiency

Parameter	Admission	Two weeks after admission	Three months after admission	Five months after admission	Seven months after admission
**Clinical data**					
Age, months	5	6	8	10	12
Weight, g	4,800	5,660	6,515	7,000	7,445
Length, cm	60	61.5	65.5	67.5	69
Head circumference, cm	39	42	43.5	45	46
Pathological clinical findings	Poor weight gain, feeding difficulties, severe pallor, muscle hypotonia, somnolence, enlarged liver and spleen	Persistence of hypotonia and mildly enlarged liver and spleen	Persistence of mild hypotonia and mildly enlarged liver and spleen	Persistence of mild hypotonia	Persistence of mild hypotonia
Pathological neurological findings	Significant delay in developmental milestones and communicative reactions	Significant delay in developmental milestones and communicative reactions	Significant delay in developmental milestones and communicative reactions	Able to remain seated with delay in other developmental milestones and communicative reactions	Able to remain seated, delay in gross motor function and language; Griffith’s scale 86 (normal values, 92–100)
**Laboratory data**					
Hb, g/dL	4.7	10	n.a.	n.a.	12.2
MCV, fL	84.2	91.3	n.a.	n.a.	81.2
White blood cell count, cells/mm^3^	4,680	9,810	n.a.	n.a.	13,410
Platelet count, cells/mm^3^	45,000	492,000	n.a.	n.a.	375,000
Peripheral blood film	Oval macrocytes, anisopoikilocytosis, hypochromia, anisochromia, and hypersegmented polymorphonuclear leukocytes	Normal	n.a.	n.a.	Normal
Bone marrow examination	Reduced number of red line cells with some megaloblasts; a small number of dysplastic megakaryocytes; dysplastic granulopoiesis with asynchronous maturation processes	n.a.	n.a.	n.a.	n.a.
Serum vitamin B12, pg/mL	57	>2,000	n.a.	n.a.	1,344
Serum folic acid, ng/mL	12.8	14	n.a.	n.a.	17.3
Serum iron, μg/dL	9	76	n.a.	n.a.	73
Serum ferritin, ng/mL	4 ng/mL	31	n.a.	n.a.	49
Blood homocysteine, μmol/L	11	5.4	n.a.	n.a.	2
Urinary methylmalonic acid, μmol/mol creatinine	281	6	n.a.	n.a.	4
*MMACHC* gene mutations	None	n.a.	n.a.	n.a.	n.a.
*Neuroimaging*					
MRI	Mild dilatation of lateral ventricles with diffuse retarded myelination, most markedly in the brainstem	n.a.	n.a.	n.a.	Persistent mild dilatation of lateral ventricles with diffuse retarded myelination, most markedly in the brainstem

MRI: magnetic resonance imaging; n.a.: not available.

In the sixth month of life, the child was weaned and green vegetables as well as fish was introduced in his diet. There was an increase in his achievement of developmental milestones, but he still showed significantly delayed neuropsychiatric development (mainly in gross motor function and language) and brain myelination seven months after the start of therapy.

## Conclusions

This case of a 5-month-old Italian infant with severe pancytopenia and neurological impairment born to a vegan mother who had received B12 supplementation during pregnancy but not during lactation seems to be particularly interesting for various reasons.

The first is the fact that it is very important to be aware that vitamin B12 and iron deficiency can frequently occur in infants born to vegan mothers (who are increasing in number in these last years) and these deficits are a preventable cause of neurodevelopmental delay. The second is the fact that the mother was treated with a multivitamin preparation that provided 2.5 μg/day of vitamin B12, an amount that is quite similar to the 2.6 μg/day recommended for pregnant women by health authorities [5]. Despite this, the infant showed clinical signs clearly attributable to vitamin B12 deficiency (such as a failure to thrive and pallor) in the first months of life, which suggests that the recommended amount of vitamin B12 is not enough to avoid the early development of disease in the infants of strictly vegetarian women. Moreover, the concomitant presence of substantial iron deficiency modified the characteristics of the anemia and may also justify several symptoms (i.e., lethargy and irritability) and signs (i.e., demyelination and poor developmental progression). Instead of significant megaloblastosis, we found red blood cells with a normal MCV, a finding that initially made it more difficult to reach a correct diagnosis. This also underlines the need to determine vitamin B12 and iron levels in all cases of otherwise unexplained severe anemia occurring in the first months of life. Finally, the MRI data confirmed the view of Lovblad *et al*. [6] that the most important neurological damage due to vitamin B12 deficiency is reduced myelination, which can persist for several months even after the start of supplementation therapy.

The possible development of severe clinical signs of vitamin B12 deficiency in infants born to undernourished mothers has been repeatedly described. A recent review of 134 cases of childhood vitamin B12 deficiency published over the last 20 years found that 69 were due to maternal B12 deficiency, and that more than 50% of these were directly related to an inadequate consumption of meat and other animal products such as that usually characterising strict vegetarians [7]. The children of women with low vitamin B12 levels during pregnancy and lactation may have smaller stores of the vitamin at birth [8], and its concentration in breast milk is likely to be low [9]. Such children tend to develop signs of vitamin B12 deficiency generally not before the fourth month of life, although neonatal cases have also been reported [10].

Childhood vitamin B12 deficiency is probably significantly more frequent than usually thought. Various studies carried out in the developing world or in countries where vegetarian diets are common have shown that a large number of mothers and children have low serum vitamin B12 levels that closely correlate with albeit mild clinical problems mainly involving the central nervous system. It has been reported that approximately one-third of low-income women and children in Guatemala have deficient (<148 pmol/L) or marginal (148–220 pmol/L) plasma vitamin B12 concentrations, and that the lowest levels are strictly related to poor growth and development [11]. In India, where people tend to be vegetarians, vitamin B12 deficiency during pregnancy is common [12], and the infants of deficient mothers are affected by a syndrome including mild developmental regression and alterations in skin pigmentation [13].

Vitamin B12 supplementation in pregnant and lactating women, and the greater use of complementary vitamin B12-rich foods in infants aged >6 months are frequently suggested strategies for reducing the risk of major clinical signs of deficiency [14,15]. However, it is difficult to implement preventive programs in the developing world as economic problems may profoundly impact the consumption of meat and other animal products. In industrialised countries, where the problem is less frequent and only vegans are at risk, a correct evaluation of the mother’s nutritional history and systematic supplementation during pregnancy and lactation can easily prevent the problem. Unfortunately, there is a lack of information concerning the optimal formulation of micronutrient supplements for pregnant women, and the need to continue their administration after delivery is not recognised in many situations in which maternal and infant health might benefit [16].

Despite a low vitamin B12 intake and significant deficiency, pregnant women generally show no related signs or symptoms because they usually consume large amounts of vegetables containing high folate concentrations that may mask the hematological effects of vitamin B12 deficiency. Furthermore, the neuropsychiatric symptoms of vitamin B12 deficiency are usually mild in adolescents and adults, and may therefore be overlooked.

On the contrary, young infants frequently have multiple deficiencies and so, particularly when they are exclusively breastfed, they develop hematological disease with anemia and (in the most severe cases) pancytopenia. As the developing central nervous system seems to be more sensitive to vitamin B12 deficiency than that of adults [17], breastfed infants born to vegan mothers may experience substantial neurological damage with developmental delay, irritability, tremor and convulsions. Furthermore, these may persist and lead to long-term cognitive and developmental delay despite adequate therapy and the complete disapperance of hematological problems [18].

As in our case, treated vitamin B12 and iron deficient infants tend to improve rapidly: hematological values quickly return to normal, and the neurological signs progressively decrease. However, in most of the severe cases described thus far [18], some neurological problems may be permanent.

In conclusion, this case underlines the importance of adequate maternal vitamin B12 intake during pregnancy by means of supplementation which, in the case of vegans, should be significantly greater than that usually given. Moreover, the supplementation should be continued during lactation in order to avoid the development of signs of deficiency that may be associated with persistent neurological problems in infancy. This case also highlights the need to consider vitamin B12 deficiency in infants with severe anemia even if their hematological parameters do not indicate megaloblastic anemia because the concomitant presence of substantial iron deficiency may modify the characteristics of the anemia.

## Consent

The patient’s parents gave their written consent to the publication of this case report.

## Abbreviation

MRI, Magnetic resonance imaging.

## Competing interests

The authors have no competing interests.

## Authors’ contributions

SG, GC and FM were responsible for making the diagnosis, treating the infant in hospital and for the follow up. SS decided the nutritional needs of the patient and his diet. NP and SE co-wrote the manuscript. All of the authors have read and approved the final manuscript.

## Pre-publication history

The pre-publication history for this paper can be accessed here:

http://www.biomedcentral.com/1471-2431/12/85/prepub
